# Systematic Review on the Worldwide Disparities in the Frequency and Results of Emergency Medical Services (EMS) and Response to Out-of-Hospital Cardiac Arrest (OHCA)

**DOI:** 10.7759/cureus.63300

**Published:** 2024-06-27

**Authors:** Syed Shahzil Parvez, Shiza Parvez, Irfan Ullah, Syed Shahrukh Parvez, Mushtaq Ahmed

**Affiliations:** 1 General Medicine, Frimley Health NHS Foundation Trust, Frimley, GBR; 2 Internal Medicine, Dr. Ruth K. M. Pfau, Civil Hospital, Karachi, PAK; 3 Accident and Emergency, Shaheed Mohtarma Benazir Bhutto Institute of Trauma (SMBBITC), Karachi, PAK; 4 Gastroenterology and Hepatology, Dr. Ziauddin Hospital, Karachi, PAK; 5 Trauma and Orthopaedics, North Devon District Hospital, Barnstaple, GBR

**Keywords:** prisma, out-of-hospital cardiac arrest (ohca), bystander interventions, bystander cpr, emergency medical service

## Abstract

This systematic analysis aimed to analyze the key patterns and tendencies regarding bystander interventions, emergency medical service (EMS) systems, dispatcher support, regional and temporal differences, and the influence of national efforts on survival rates in out-of-hospital cardiac arrest (OHCA). The studies published between 2010 and 2024 examining outcomes of OHCA, interventions by bystanders, and variables linked to OHCA were included in this research. The inclusion process was done under Preferred Reporting Items for Systematic Reviews and Meta-Analyses (PRISMA), where publications (n = 24) from various geographical locations, including a wide range of research methodologies, were included for this research. The thematic analysis used for the data analysis shows that bystander cardiopulmonary resuscitation (CPR) enhances the chances of survival. The effectiveness of the EMS system, the assistance offered by dispatchers, and the inclusion of doctors in ambulance services are essential components in the management of OHCA. Regional and temporal variations highlight disparities in resuscitation protocols, emphasizing the need for adaptable approaches. Observations from statewide endeavors emphasize the impact of these activities in fostering a culture of prompt bystander intervention. This systematic review presents a comprehensive analysis of research conducted globally, providing a thorough insight into the variables that influence survival rates in instances of OHCA. The review recognizes the importance of bystander CPR and effective EMS services, while also bringing novel perspectives, such as gender disparities and geographical variations that contribute to the existing body of research. Despite possible variances in the studies and biases, the findings underscore the need for tailored therapies and ongoing research to optimize strategies for controlling OHCA and improving survival rates.

## Introduction and background

Out-of-hospital cardiac arrest (OHCA) can be a severe and frightening situation requiring immediate assistance [1]. A well-organized “chain of survival” should be adopted for the survival of life. For individuals out of the hospital, the “chain of survival” entails bystander cardiopulmonary resuscitation (CPR), automatic external defibrillators, and presentation in emergency medical services (EMS) [2]. One paper presented the significant public health problem of OHCA. Worldwide, it occurs in around 55 adults per 100,000 person-years on average, and fewer than one in 10 of these people survive [3]. There are many reasons behind undertaking a systematic review and meta-analysis regarding the variability in the frequency and outcomes of EMS-witnessed OHCA worldwide. The reason/reasons are multifaceted. The quality of care that the patient receives before reaching a hospital plays a significant role in determining whether they survive and improve their health [4]. Looking at the differences in how EMS responds and what happens on a world level can show patterns, differences, and maybe even ways to manage OHCA better. There are also differences in the facilities, finances, and pre-hospital care skills that affect how well OHCA events are responded to.

Policymakers, healthcare professionals, and academics can learn a lot about how structural factors influence the results of OHCA by looking at these changes worldwide. This can make it easier to develop focused ways to improve EMS. Moreover, knowing a lot about OHCA trends worldwide can help you figure out the best ways to do things in places where they work well. These can then be changed and used in areas where they also do not work. Therefore, sharing what you know or learn is valuable. It can help create standardized methods and treatments, improving survival rates while decreasing the long-term health issues associated with OHCA.

This research study aims to determine why the number and outcomes of OHCA cases presented to EMS vary in different countries. This review attempts to analyze datasets for trends and factors that lead patients in varied application areas to obtain poor outcomes. This systematic review helps to fill this gap in the literature by comparing and contrasting the incidence of EMS-treated OHCA at a global level. No study to date has attempted to explore these variations together at a global level. We sought to offer a review of this recent literature that offers an overview of the various disparities found and what these disparities mean.

## Review

Research methodology

Research Design and Data Collection

Systematic review and meta-analysis follow the Preferred Reporting Items for Systematic Reviews and Meta-Analyses (PRISMA) guidelines [5]. In this study, a comprehensive search strategy was developed to explore worldwide differences in OHCA understood by EMS. We searched three significant databases: PubMed, Medline, and Web of Science. Subsequently, the method involved using the appropriate keywords integrated with suitable MeSH phrases ("out-of-hospital cardiac arrest," "emergency medical services," and their combination with “global variation incidence,” "outcome," "observational studies," and “quantitative studies”).

Inclusion Criteria

The systematic review will consider for inclusion papers that meet specific criteria: research from which human subjects who experienced OHCA and were treated by EMS are exposed. The incidence and impact of OHCA will be the prime concern of such research, considering survival, neurological implications, and other associated indicators. Eligible studies could be quantitative, observational, or clinical trials that measure OHCA attended by EMS. The review only considered documents prepared in English and addressing worldwide contexts to ensure that the analysis finds geographically diverse perspectives between 2010 and 2024. This comprehensive approach will robustly assess the global heterogeneity in incidence and sequelae associated with EMS-attended OHCA.

Exclusion Criteria

Furthermore, research that did not meet the criterion for specific inclusion was also rejected. For instance, studies that fall outside the scope and relevance of OHCA, research not involving EMS systems, and research based solely on in-hospital cardiac arrest (IHCA) data; all in all, the external validity of the clinical trial was high. However, to keep up with the systematic review’s integrity, works published other than English language and before 2010 were not considered, along with conference summary papers and editorials/letters/case reports. To uphold the review’s focus on complete research, any paper failing to provide ample detail regarding the prevalence or outcomes of EMS-witnessed OHCA was not included. The purpose of inclusion criteria is to strengthen the selection process, so this systematic review includes only those studies that meet determined criteria requirements.

Screening Process

The screening procedure began with the independent evaluation of titles and abstracts by two experienced reviewers from subjects related to this review and inexperience within systematic reviews. The selected publications were then accessed to determine if they focused on OHCA-affected human participants treated by the EMS and reported outcomes surrounding the incidence, survival, or complication rates. Exclusion criteria were applied to exclude studies that did not meet the specific needs of the review, such as papers with a primary judgment of cardiac arrest within a hospital setting or those without relevant data available for OHCA attended by EMS.

After the first screening, the full papers of potentially relevant research were retrieved and screened in detail based on inclusion criteria. Any discrepancies or disagreements between the two reviewers at any stage were resolved through discussion or, when needed to solve deadlock scenarios, the intake of a third reviewer. In summary, the extensive selection procedure was designed to encompass high-quality and contextually significant studies and considerably impact the overall evidence synthesis of EMS-witnessed OHCA across the globe.

Data Mining

Data mining is scanning all the extracted studies to extract relevant data. We developed a standardized data extraction form to ensure that retrieval of pertinent data points was consistent and comprehensive. Two reviewers collected the information independently, and the results were cross-validated to remove possible errors or biases. Collected data also contain essential parts, such as research characteristics (e.g., author and year of creation), participants' descriptions and information about the geography, methodology used in the study, and particular OHCA features. The inquiry, he says, carefully collected data on characteristics including the incidence rates of OHCA witnessed by EMS movement to clinic, endurance, and results like neurological deficiency, among others. Attempts to standardize data that were presented in different formats or even measurements because it was necessary to compare the information effectively. The data extraction approach also allowed for gathering relevant information about potential confounders that may lead to bias, contributing to a better understanding of the global heterogeneity of EMS-witnessed OHCA. Consensus talks resolved all discrepancies and contradictions in the data collection.

Quality Assessment

A systematic and transparent assessment of the quality of selected studies was conducted to maintain reliability and validity. Appraisal of the study designs included have been scored based on validated tools. In particular, the Newcastle-Ottawa Scale (NOS) was used for observational studies to evaluate methodological quality based on criteria that referenced selection, comparability, and outcome (Table 1).

**Table 1:** Quality assessment of the included studies

Author/Year	Selection	Comparability	Outcome	Total stars (out of 9)
Adielsson A et al. (2011)[6]	★★★	★★★	★★★★	9
Blewer AL et al. (2018) [7]	★★★	★★	★★★	8
Fothergill RT et al. (2013) [8]	★★★	★	★★★	7
Gaieski DF et al. (2017) [9]	★★★	★★	★★★	8
Hagihara A et al. (2014) [10]	★★★	★★★	★★★	9
Hagihara A et al. (2018) [11]	★★★	★★★	★★★	9
Hasselqvist-Ax I et al. (2015) [12]	★★★	★★★	★★★	9
Iwami T et al. (2012) [13]	★★★	★	★★★	7
Iwami T et al. (2015) [14]	★★★	★	★★★	7
Kaneko H et al. (2017) [15]	★★★	★★★	★★★	9
Kitamura T et al. (2012) [16]	★★★	★★★	★★★	9
Kitamura T et al. (2014) [17]	★★★	★★★	★★★	9
Lai H et al. (2015) [18]	★★★	★★	★★★	8
Mathiesen WT et al. (2018) [19]	★★★	★★★	★★★	9
May S et al. (2018) [20]	★★★	★★	★★★	8
Nehme Z et al. (2016) [21]	★★★	★	★★★	7
Ong ME et al. (2015) [22]	★★★	★★	★★★	8
Rajan S et al. (2016) [23]	★★★	★★	★★★	8
Ro YS et al. (2015) [24]	★★★	★★★	★★★	9
Shao F et al. (2014) [25]	★★★	★★	★★★	8
Sondergaard KB et al. (2019) [26]	★★★	★★★	★★★	9
Tanaka H et al. (2018) [27]	★★★	★★★	★★★	9
Wissenberg M et al. (2013) [28]	★★★	★★★	★★★	9

Data Synthesis and Analysis

The study used a meta-analysis to analyze and explain the patterns and differences discovered in various geographical regions. Thematic analysis entailed the systematic arrangement and analysis of results generated from individual research. This technique identifies and investigates critical themes concerning occurrence rates, survival outcomes, and long-term neurological effects. The focus was clarifying the contextual elements contributing to global variances in OHCA outcomes witnessed by EMS. These factors include variations in healthcare infrastructure, pre-hospital care regimens, and demographic characteristics.

Study Selection

**Figure 1:**
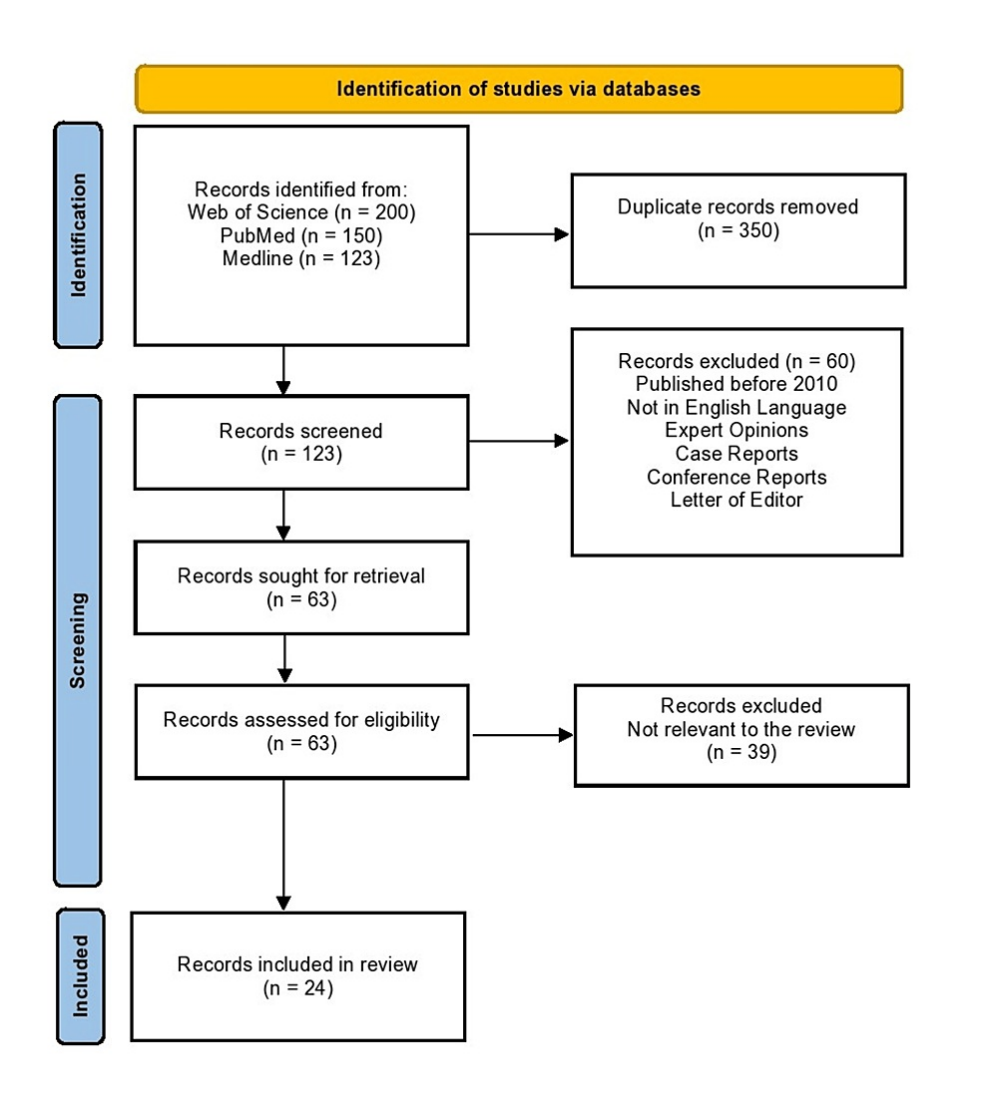
Preferred Reporting Items for Systematic Reviews and Meta-Analyses (PRISMA) flowchart

Figure 1 presents the research selection, identification, and inclusion procedure using the PRISMA flow diagram. First, 473 articles were obtained from the Medline, PubMed, and Web of Science databases. After eliminating 350 duplicate articles, we removed 60 items by considering the titles and abstracts. This included 60 studies on nonhuman subjects and papers categorized as reviews, editorials, letters, guidelines, or case reports. Sixty-three articles were chosen for additional evaluation of their whole texts. After obtaining the complete text for a thorough review, 35 papers were removed due to their inclusion of pediatric patients in the study group, their primary focus on the neurological survival rate, or their duplication as several publications employing the same study population. In the current meta-analysis, a total of 28 papers were ultimately incorporated.

Thematic analysis

Bystander CPR (BCPR) and Survival

Several studies have shown that BCPR is crucial in determining survival outcomes. The survey undertaken by Adielsson et al. (2011) in Sweden provided an 18-year outlook and showed a significant rise in survival rates linked to BCPR, namely, in out-of-hospital shockable arrhythmias. The study highlights that female gender is a predictor for better outcomes, which suggests that there may be differences in the application of BCPR based on demographic characteristics [6]. Similarly, Blewer et al. (2018) explored the gender gap in public settings of bystander CPR delivery. They addressed the importance of ensuring equal opportunities even when providing means with the highest saving potential. The research further emphasized the importance of involving the public and carrying out CPR teaching programs to include individuals from both genders [7]. Moreover, according to Hasselqvist-Ax et al. (2015), CPR should be administered after OHCA; an increased survival rate has been linked to the same [12]. “And this topic highlights the critical need for bystander resuscitation attempts because that is what significantly increases survival rates among patients who have a cardiac arrest event out-of-hospital,” said Shailja. Table 2 results underscore the importance of public awareness campaigns, training, and community participation in making the life-saving measures effective in all cases of cardiac crisis.

**Table 2:** Study's characteristics OHCA: out-of-hospital cardiac arrest

Author	Study design	Location	Total population	Origin of OHCA	CPR type	Witnessed or not	Outcomes	Survival at discharge/Incidence
Adielsson et al. (2011) [6]	Observational (Retrospective Cohort)	Sweden	7,187	Cardiac aetiology	Bystander CPR	Witnessed	Survival to admission, 1-month survival	Survival rate at 1 month is 976
Blewer et al. (2018) [7]	Observational (Retrospective Cohort)	US and Canada	19,331	Non-traumatic	Bystander CPR	Witnessed, unwitnessed	Return of spontaneous circulation, survival to discharge	Survival rate at 1 month is 2261
Fothergill et al. (2013) [8]	Observational (prospective Cohort)	UK	21,020	Cardiac aetiology	CPR	Witnessed	Survival to discharge	Survival rate at 1 month 497/ incidence 117.8 per 100,000
Gaieski et al. (2017) [9]	Observational (Retrospective Cohort)	US	5,198	Non-traumatic	CPR	Witnessed, unwitnessed	Return of spontaneous circulation	Survival rate at 1 month is 401/ incidence 81.5/100,000
Hagihara et al. (2014) [10]	Observational (Prospective Cohort)	Japan	619,928	All patients	Emergency medical services CPR	Witnessed, unwitnessed	Return of spontaneous circulation, 1-month survival	Survival rate at 1 month is 1917
Hagihara et al. (2018) [11]	Observational (Retrospective Cohort)	Japan	87,400	Cardiac etiology	Bystander CPR	Witnessed, unwitnessed	Return of spontaneous circulation, 1-month survival	Survival rate at 1 month is 2345
Hasselqvist-Ax et al. (2015) [12]	Randomised Controlled Trial	Sweden	15,512	Cardiac, all patients	Bystander CPR	Witnessed	1-month survival	Survival rate at 1 month is 1551
Iwami et al. (2012) [13]	Observational (Nationwide cohort)	Japan	1,376	Cardiac aetiology	Bystander CPR	Witnessed	Return of spontaneous circulation, 1-month survival	Survival rate at 1 month is 492
Iwami et al. (2015) [14]	Observational (Nationwide cohort)	Japan	816,385	All patients	Bystander CPR	Witnessed, unwitnessed	Return of spontaneous circulation, 1-month survival	Survival rate at 1 month is 33038
Kaneko et al. (2017) [15]	Observational (Prospective Cohort)	Japan	44,706	All patients	Bystander CPR	Witnessed	Return of spontaneous circulation, 1-month survival	Survival rate at 1 month is 4023
Kitamura et al. (2012) [16]	Observational (Nationwide cohort)	Japan	547,153	All patients	Bystander CPR, CPR	Witnessed	Return of spontaneous circulation, 1-month survival	Survival rate at 1 month is 8804
Kitamura et al. (2014) [17]	Observational (Nationwide cohort)	Japan	10,876	Cardiac aetiology	CPR	Witnessed	Return of spontaneous circulation, survival to admission, 1-month survival	Survival rate at 1 month is 522
Lai et al. (2015) [18]	Observational (Retrospective Cohort)	Singapore	5,453	Cardiac etiology	CPR	Witnessed, unwitnessed	Survival to admission	Survival rate at 1 month is 394
Mathiesen et al. (2018) [19]	Observational (Comparative)	Norway	1,138	Cardiac etiology	Bystander CPR	Witnessed, unwitnessed	Survival to discharge	Survival rate at 1 month is 214/ Incidence 47 per 100, 000
May et al. (2018) [20]	Observational (Retrospective Cohort)	US	2,359	Non-traumatic	CPR, bystander CPR, emergency medical services CPR	Witnessed, unwitnessed	Survival to admission, survival to discharge	Survival rate at 1 month is 121
Nehmea et al. (2016) [21]	Observational (Prospective Cohort)	Australia	1,035	Cardiac etiology	Emergency medical services CPR	Witnessed	Return of spontaneous circulation, survival to admission, survival to discharge	Survival rate at 1 month is 382
Ong et al. (2015) [22]	Observational (Retrospective Cohort)	Japan, Korea, Malaysia, Singapore, Thailand, Taiwan, UAE	66,780	Cardiac etiology	Bystander CPR	Witnessed	Survival to admission, survival to discharge	Survival rate at 1 month is 5676
Rajan et al. (2016) [23]	Observational (Retrospective Cohort)	Denmark	7,623	Cardiac etiology	Bystander CPR	Witnessed, unwitnessed	Return of spontaneous circulation, 1-month survival	Survival rate at 1 month is 623
Ro et al. (2015) [24]	Observational (Retrospective Cohort)	Japan, Korea	36,292	Cardiac etiology	CPR	Witnessed	Survival to admission, survival to discharge	The survival rate at 1 month is 2913/ Incidence 39.1 per 100, 000
Shao et al. (2014) [25]	Observational (Retrospective Cohort)	China	9,897	Cardiac etiology	Bystander CPR, CPR	Witnessed, unwitnessed	Return of spontaneous circulation, survival to admission, survival to discharge	Survival rate at 1- month is 22/Incidence 80.6 per 100, 000
Sondergaard et al. (2019) [26]	Observational (Prospective Cohort)	Denmark	25,505	Cardiac etiology	Bystander CPR	Witnessed, unwitnessed	1-month survival, 1-year survival	Survival rate at 1- month is 2281
Tanaka et al. (2017) [27]	Observational (Prospective Cohort)	Japan, Korea, Malaysia, Singapore, Thailand, Taiwan, Emirates	Not specified	Non-traumatic	CPR	Witnessed, unwitnessed	Survival to admission	Not specified
Wissenberg et al. (2013) [28]	Observational (Nationwide Cohort)	Denmark	29,111	Cardiac etiology	CPR	Witnessed, unwitnessed	Survival to admission, 1-month survival, one-year survival	Survival rate at 1- month is 9316/ incidence is 34.6 per 100, 000

EMS Systems and Survival

Characterizing and analyzing EMS systems is essential in determining the differences in outcomes between survivors of OHCA. In a study undertaken by Mathiesen et al. in 2018, prison quality from the perspectives of Norwegian inmates was seen as less important in contributing to the future offending than longitudinal data or reconviction rates indicate. A common approach for EMS implementation across various countries is necessary, as per the European Emergency Data EED Project [19]. Finally, this study underlines the importance of effective emergency response systems and how they may offer a margin for survival among patients facing geographical differences in healthcare facilities. Furthermore, Ong et al. (2015), who studied the impact of OHCA in seven Asian countries, however, conducted a more thorough exploration of this topic. It highlights the differences in findings and perhaps the different influences of separate EMS systems across various cultures and geographies. These findings emphasize adapting resuscitation protocols based on geographical features [22]. This scene reinforces the belief that all life-saving efforts depend on how effectively EMS systems are implemented. It also highlights the importance of prospective efforts to improve the infrastructure and protocols related to pre-hospital care to yield optimal outcomes in cases of OOHCA worldwide.

The Presence of a Doctor and Assistance From a Dispatcher

It is also noted that the presence of a physician and dispatcher assistance remarkably allows for a higher survival rate. Hagihara et al. (2014) argued that having a doctor in an ambulance increases survival rates. It stresses the importance of having highly qualified medical staff at this early stage of resuscitation. In the post-resuscitation phase, any patient successfully resuscitated is placed on ventilation and carefully managed in an ICU setting. Furthermore, Hagihara et al. (2018) examined the benefits of dispatcher-assisted bystander CPR and found a relationship between this type of care and increased survival after OHCA. This chain of survival is incomplete without the dispatcher because it maintains support and provides assistance until professional care arrives [11]. The current topic discusses the importance of proper collaboration between emergency dispatch systems on the one hand and responders present at the scene of OHCA events, as well as immediate involvement of medical expertise in resuscitation efforts on the other for improving patient outcomes.

Geographical and Time-Based Patterns

Investigating the patterns of regional and temporal survival rates is a crucial topic to address regarding OHCA analysis. Significantly, research such as this is critical to understanding how resuscitation methods adapt and how these shifts impact patient survival. One such study, which was conducted by Ro et al. (2015), tested the changes in survival rates over time in urban areas. They compared two metropolitan areas to identify how they changed. The results of this study could provide future contributors with a more thorough understanding regarding the advancement of resuscitation efforts. Temporal fluctuations are shown in this study, indicating that the survival rate could be potentially influenced by further improvement of EMS, public awareness, or other factors [24]. Kitamura et al. (2012) analyzed survival rates of OHCA nationwide in Japan, focusing on the national contribution to improve the outcome of resuscitation [16]. The studies emphasized that survival patterns in OHCA were ever-changing, changing with the regional factors, cultural subtleties, and refinement of resuscitation techniques. Recognizing and adapting to these patterns is necessary to devise tailored interventions or implement and adopt policies that equitably improve survival across distinct geographical areas and throughout time.

National Programs and Efforts Aimed at Improving the Quality of Healthcare Services

The focus on improving patient outcomes reflects national efforts to increase cardiac arrest management and the quality of EMS care. This study by Wissenberg et al. (2013) rigorously explored the association between national programs and rates of bystander participation, which ultimately influenced patient survival rates after OHCAs. Thus, this research underlines the fact that there is a straight correlation between national attempts to implement resuscitation and efforts aimed at higher bystander engagement, which makes overall country-level policies effective [28]. Rajan et al. (2016) analyzed the association of bystander CPR with survival outcomes and compared ambulance response times after an OHCA. The paper uses 10-year data from the Victorian Cardiac Arrest Ambulance Registry to underscore how comprehensive data is integral to evaluating the quality of EMS [23]. This requires coordinated nationwide measures and continued quality improvement to teach a fast, influential culture of bystander intervention. In the end, these things help increase the chances of survival for someone who has a cardiac arrest. Hence, the results stress the importance of consistent methodologies, data-based regulations, and continuous efforts to improve the national quality of emergency care.

Discussion

The research encompassed in this systematic review collectively offers insights into diverse parameters that influence survival outcomes in cases of OHCA. The identified themes, ranging from the importance of bystander CPR, the effectiveness of EMS systems, dispatcher assistance, regional worthy-of-consideration, and temporal trends with the impact of national initiatives on quality of care overall, offer an all-around understanding regarding dynamics in OHCA life management. Data from previous systematic studies reveal commonalities and new findings.

Prior systematic evaluations have consistently highlighted the importance of CPR initiated by bystanders in enhancing survival rates after OHCA [29,30]. The findings of Adielsson et al. (2011) and Blewer et al. (2018) support and emphasize the existence of gender discrepancies, underscoring the necessity for treatments that are specifically tailored to address these disparities [6,7]. The current analysis expands on existing knowledge by investigating the influence of bystander CPR on particular populations, such as those experiencing shockable arrhythmias and witnessed events, in a manner consistent with previous research.

The analysis of EMS systems and their influence on survival outcomes mirrors the emphasis of previous evaluations. Mathiesen et al. (2018) significantly contribute to this topic, highlighting the significance of implementing consistent methods across various locations [19]. This is consistent with other research, highlighting the importance of internationally flexible EMS solutions. Nevertheless, Ong et al. (2015) presented a novel perspective by examining outcomes in many Asian nations, providing valuable insights into the varied cultural and geographical factors that impact survival rates of OHCA [22]. This unique viewpoint enhances the existing knowledge base by providing vital information.

The need for dispatcher support and a physician's presence during OHCA has been acknowledged as crucial elements of the chain of survival. The investigations conducted by Hagihara et al. in 2014 and 2018 confirm these concepts, emphasizing the beneficial influence of healthcare professionals who are present at the site or offer remote guidance [9,10]. This regularity strengthens the significance of incorporating medical proficiency into first resuscitation endeavors.

Investigating regional and temporal patterns expands on the knowledge that resuscitation techniques change over time and can differ among various geographical areas. Ro et al. (2015) contributed significantly to this topic by highlighting the ever-changing nature of outcomes in cases of OHCA [24]. These studies provide new information on current trends and differences in resuscitation tactics, which align with earlier research. They also emphasize the importance of continuously adapting these strategies.

National initiatives have been acknowledged as significant in enhancing survival outcomes by improving care quality. Wissenberg et al. (2013) and Rajan et al. (2016) support this viewpoint by highlighting the direct relationship between systematic, nationwide strategies and higher levels of bystander intervention [23, 28]. This underscores the significance of implementing comprehensive, nationwide efforts to cultivate a culture that encourages immediate and efficient bystander intervention.

Limitations and Strengths

This systematic review has numerous limitations that should be taken into account. Initially, it is essential to note that the included studies exhibit significant variations in methodology, demographics, and geographic locations. The existence of heterogeneity in data will have two consequences: first, it may cause problems during the synthesis and combination of results, and second, it will limit generalization to a broader population. Moreover, publication bias may occur, leading to a source of bias in the appraisal of evidence on the effect of interventions as a whole. Furthermore, regional bias might be another source of influence in this review because studies mainly focus on particular areas, which can weaken the results that can be generalized on a global stage. When specific datasets contain incomplete data, it might challenge one’s ability to conduct a comprehensive subgroup analysis and consider all relevant confounders, affecting the results' precision.

However, despite all these limitations, the systematic review has several incredible strengths. Combining all studies from different centers in various geographical regions contributes to the broadened and inclusive understanding of OHCA management. This ability may aid in recognizing any potential regional discrepancies and gauging the efficacy of therapies among different populations. This systematic presentation of findings enhanced the clarity and allowed a comprehensive examination of how bystander action, EMS systems, or other variables influenced survival rates. An in-depth review of the multiple elements influencing OHCA outcomes offers an exhaustive snapshot regarding the current state of this science. In this review, we present our original results and conclusions based on the data collected in several studies examining differences between women and men, specific modalities of CA presenting in characteristic episodes of life-threatening heart rhythms or localisations, and geographical distribution. Therefore, the systematic review’s united strengths serve as an ultimate source for understanding OHCA’s complex dynamics regardless of challenges associated with pooling data information from multiple sources.

## Conclusions

The current systematic review comprehensively examines multiple determinants impacting survival probability in OHCA cases. Given the synthesis of data from various geographical areas and thematic analysis, it becomes apparent what the key factors are behind good OHCA management. One such tenet is bystander CPR, supported by a plethora of research that has continually shown the positive impact on survival from this intervention. In this regard, it is significant that the efficiency of EMS systems involving and reducing response times to focus within 60 minutes of being initiated by a physician-informed name via dispatcher assistance also has an essential part in maintaining continuity of care.
